# Supplementary material for: Genetic Biomarkers of Sorafenib Response in Patients with Hepatocellular Carcinoma
Source: Int J Mol Sci. 2024 Feb 12;25(4):2197. doi: 10.3390/ijms25042197 (PMC10888718; doi:10.3390/ijms25042197)
Supplement: Supplementary file 1 [file ijms-25-02197-s001.zip › ijms-2847640-supplementary.pdf]

Supplementary Table S1. Distribution of demographic characteristics and clinical parameters of patients (n = 34).

**Age** (yr; mean ± SD)<br>(range: 57 - 89)		
		73 ± 6
**Gender** (n; %)		
	female	10 (29)
	male	24 (71)
**Etiology** (n; %)		
	HCV	19 (56)
	HBV	6 (18)
	alcoholic	3 (9)
	cryptogenetic/metabolic	6 (18)
**BCLC stage** (n; %)		
	A	3 (9)
	B	21 (62)
	C	10 (29)
**Child-Pugh class** (n; %)		
	A	26 (76)
	B	6 (18)
	C	2 (6)
**AFP** (n; %)<br>(range: 0.2 - 20,000)		
	≥ 400	4 (12)
	< 400	30 (88)
**Prior surgery/locoregional treatments** (n; %)		
	yes	22 (65)
	no	12 (35)
**Portal invasion** (n; %)		
	yes	6 (18)
	no	28 (82)
**Sorafenib dose reduction** (n; %)		
	yes	15 (44)
	no	19 (56)

AFP = Alpha-fetoprotein; BCLC = Barcelona Clinic Liver Cancer.

Supplementary Table S2. Genetic risk score in responder and non-responder patients (n = 23).

				**responders (means ± SD)**	**non-responders (means ± SD)**	***p***
**5 variants (score 0 -10)**						
rs4604006 (VEGF-C)	rs12434438 (HIF-1A)	rs183574 (SLC22A14)	rs6811453 (ADH1A)	6.00 ± 0.81	4.37 ± 1.36	***0.008***
